# Effective behaviour change techniques in smoking cessation interventions for people with chronic obstructive pulmonary disease: A meta-analysis

**DOI:** 10.1111/bjhp.12071

**Published:** 2013-10-30

**Authors:** Yvonne K Bartlett, Paschal Sheeran, Mark S Hawley

**Affiliations:** 1 School of Health and Related Research, University of SheffieldUK; 2 Department of Psychology, University of SheffieldUK

**Keywords:** smoking cessation, chronic obstructive pulmonary disease, behaviour change techniques, meta-analysis, systematic review

## Abstract

**Purpose:**

The purpose of this study was to identify the behaviour change techniques (BCTs) that are associated with greater effectiveness in smoking cessation interventions for people with chronic obstructive pulmonary disease (COPD).

**Methods:**

A systematic review and meta-analysis was conducted. Web of Knowledge, CINAHL, EMBASE, PsycINFO, and MEDLINE were searched from the earliest date available to December 2012. Data were extracted and weighted average effect sizes calculated; BCTs used were coded according to an existing smoking cessation-specific BCT taxonomy.

**Results:**

Seventeen randomized controlled trials (RCTs) were identified that involved a total sample of 7446 people with COPD. The sample-weighted mean quit rate for all RCTs was 13.19%, and the overall sample-weighted effect size was *d*_*+*_* *= 0.33. Thirty-seven BCTs were each used in at least three interventions. Four techniques were associated with significantly larger effect sizes: *Facilitate action planning/develop treatment plan*, *Prompt self-recording*, *Advise on methods of weight control*, and *Advise on/facilitate use of social support*. Three new COPD-specific BCTs were identified, and *Linking COPD and smoking* was found to result in significantly larger effect sizes.

**Conclusions:**

Smoking cessation interventions aimed at people with COPD appear to benefit from using techniques focussed on forming detailed plans and self-monitoring. Additional RCTs that use standardized reporting of intervention components and BCTs would be valuable to corroborate findings from the present meta-analysis.

**Statement of contribution:**

***What is already known on this subject?*** Chronic obstructive pulmonary disease (COPD) is responsible for considerable health and economic burden worldwide, and smoking cessation (SC) is the only known treatment that can slow the decline in lung function experienced. Previous reviews of smoking cessation interventions for this population have established that a combination of pharmacological support and behavioural counselling is most effective. While pharmacological support has been detailed, and effectiveness ranked, the content of behavioural counselling varies between interventions, and it is not clear what the most effective components are.

***What does this study add?***

- Detailed description of ‘behavioural counselling’ component of SC interventions for people with COPD.
- Meta-analysis to identify effective behaviour change techniques tailored for this population.
- Discussion of these findings in the context of designing tailored SC interventions.

## Background

Chronic obstructive pulmonary disease (COPD) is a term used to describe progressive, non-reversible airflow obstruction (Department of Health [DoH], 2005). Approximately 80% of cases are linked to smoking (DoH, 2005) with the other 20% due to a combination of environmental and genetic factors (National Institute for Health and Clinical Excellence [NICE], 2010). In 2004, COPD was estimated to cost the National Health Service (NHS) £800 million in direct care costs and was responsible for 24 million lost work days (DoH, 2005). The prevalence and costs associated with COPD are expected to rise in the coming years (Parker & Eaton, 2012), and by 2020, it is estimated that COPD will be the third leading cause of death worldwide (World Health Organisation [WHO], 2002).

There is no cure for COPD (DoH, 2005). To date, the only intervention found to slow the decline in lung functioning is smoking cessation (Anthonisen *et al*., 1994). Current best practice advises that people with COPD be encouraged to quit smoking and given all necessary psycho-social and/or pharmacological support to do so (NICE, 2010). Nevertheless, the proportion of people with COPD continuing to smoke has been estimated between 32.8% and 70% (Baron, 2003; Vozoris & Stanbrook, 2011) and could be rising (Vozoris & Stanbrook, 2011). The current advice to physicians in the United Kingdom and the United States is that people with COPD should be given advice at every opportunity and, if the person is agreeable, should be referred to a local smoking cessation service (NICE, 2010; Parker & Eaton, 2012). In the United Kingdom, this is the NHS Stop Smoking Services (SSS). Target quit rates for the NHS SSS are between 35% and 70% (Willis, 2008). However, the SSS are not specifically designed for people with COPD. Evidence suggests that smokers with COPD have greater dependence on nicotine than those with normal lung functioning (Jiménez-Ruiz *et al*., 2001) and find it harder to quit (Tashkin & Murray, 2009). Continued smoking by people with COPD increases hospital admissions and negatively affects morbidity and mortality (Global Initiative for Chronic Obstructive Lung Disease [GOLD], 2011). In a recent simulation, it was estimated that continued smoking by people with COPD in England alone would result in costs of £1,657 million over a 10-year time period and that smoking cessation was cost–effective regardless of disease stage (Atsou, Chouaid, & Hejblum, 2011). Despite the health and economic benefits of encouraging smoking cessation in this population, there is relatively little evidence of smoking cessation interventions that are tailored for this group (Parker & Eaton, 2012).

### Rationale for the present review: Identifying effective behaviour change techniques

Previous systematic reviews addressing smoking cessation in people with COPD have concluded that a combination of stop smoking medication (SSM) and non-pharmacological approaches offers the most effective smoking cessation intervention for people with COPD. This finding has been supported by meta-analyses (van der Meer, Wagena, Ostelo, Jacobs, & van Schayck, 2003; Strassmann *et al*., 2009), economic modelling (Hoogendoorn, Feenstra, Hoogenveen, & Rutten-van Molken, 2010), and narrative review (Parker & Eaton, 2012; Warnier, Riet, Rutten, Bruin, & Sachs, 2012). However, although the SSM components have been ranked in terms of effectiveness (Strassmann *et al*., 2009), the efficacy of the non-pharmacological components (typically referred to as ‘behavioural counselling’) has not previously been assessed. What constitutes ‘behavioural counselling’ varies considerably between interventions (Michie & Abraham, 2008). Parker and Eaton (2012) suggest that counselling ‘should assist in motivating the smoker to quit smoking and developing skills and strategies to cope with nicotine withdrawal, and …should also help the smoker identify cues and situations that would lead to temptation or pressure to smoke’ (p. 161), although they did not describe which of the existing interventions contain these elements, or their potential relationship with intervention outcomes.

Abraham and Michie (2008) developed a taxonomy of the ‘active ingredients’ or behaviour change techniques (BCTs) used in behavioural interventions to improve comparisons between studies and enable conclusions about the efficacy of specific intervention components (Abraham & Michie, 2008). Michie and colleagues produced a taxonomy of the BCTs used in smoking cessation studies (Michie, Churchill, & West, 2011; Michie, Hyder, Walia, & West, 2011). Michie, Hyder, *et al*.'s (2011) taxonomy contains 53 specific BCTs categorized into groups according the function they perform (Table S1). Techniques coded with a ‘B’ have a specific focus on behaviour and are split into ‘BM’ (which address motivation) and ‘BS’ (which focus on self-regulatory capacity and skills). Techniques coded with an ‘A’ promote adjuvant activities and an ‘R’ focus on more general aspects of the interaction; ‘RD’ describes aspects of delivery, ‘RI’ codes for aspects of information gathering and ‘RC’ for general communication (Michie, Hyder, *et al*., 2011). These groups outline the target areas for smoking cessation counselling. However, within these codes, the taxonomy defines specific BCTs used to achieve these targets (see Table S1 for examples). This taxonomy has been used to classify existing interventions and services for the general population of smokers (West, Walia, Hyder, Shahab, & Michie, 2010), the content of a text message-based intervention for smoking cessation (Michie, Free, & West, 2012), and smoking cessation interventions during pregnancy (Lorencatto, West, & Michie, 2012). This method has not heretofore been applied to smoking interventions among people with COPD.

The purpose of this review is to identify which BCTs are associated with more effective smoking cessation interventions for people with COPD. Discovering effective BCTs could guide the development of future interventions tailored to the COPD population, to ensure maximum impact on cessation rates.

## Methods

### Search strategy

The present review was part of a larger review of behaviour change interventions in people with COPD in which smoking, exercise, and breathing training behaviours were investigated. Briefly, the search strategy comprised of COPD terms AND intervention/behaviour terms AND smoking terms OR exercise terms OR breathing training terms (for the full strategy, see Appendix 1 2008). The full search strategy (optimized for each database) was run in CINAHL, MEDLINE, PsycINFO, Web of Knowledge (all databases), and EMBASE. Articles that cited, or were cited by, included studies, and applicable reviews were assessed. A reduced search (COPD AND behav$) was conducted in PASCAL, ESTAR, AMED, and the Applied Social Sciences Index and Abstracts. The search was last updated on 27 December 2012.

### Inclusion and exclusion criteria

Papers were included if (1) smokers with a diagnosis of COPD were participants, (2) a randomized controlled trial (RCT) of an intervention that aimed to alter participants’ behaviour was reported, and (3) a measure of smoking cessation was reported. Unpublished papers and papers not written in English were excluded.

### Outcome definitions

The outcome of interest was smoking cessation (quit rate), measured by either point prevalence (PP) or continuous abstinence (CA) measures. PP measures smoking status at a specific point in time, or for a period immediately before this point; typically these measures assess whether or not the person has smoked in the last 7 days. CA measures sustained abstinence over a longer period of time, with smoking status measured on two or more occasions. Both CA and PP can be assessed using self-report, biochemical validation, or both.

### Quality assessment

Study quality was assessed using the Delphi list (Verhagen *et al*., 1998). A score of five or greater indicates a ‘high-quality study’ according to a related Cochrane review (van der Meer *et al*., 2003). Power and attrition rates were also calculated for each study.

### Coding of interventions

Interventions were coded according to a 53-item taxonomy that is specific to smoking cessation (Michie, Churchill, *et al*., 2011). To ensure comprehensive coding of the interventions, authors were contacted for any additional materials such as protocols or training materials. All English language resources provided were coded in addition to the publication. Intervention descriptions were coded by a researcher familiar with the taxonomy who had undergone training in the use of BCT taxonomies. Fifteen of the 17 interventions were further independently coded by an expert in using this taxonomy. Initial agreement between the two coders was 89.31% with a kappa coefficient of .7. This represents ‘substantial agreement’ (Landis & Koch, 1977). Disagreements were resolved by discussion.

The BCT taxonomy contains several codes related to SSM, namely *Advise on stop smoking medication*, *Adopt appropriate local procedures to enable clients to obtain free medication,* and *Ask about experiences of stop smoking medication that the smoker is using*. However, these codes do not differentiate between SSM being a prescribed, integral part of the intervention, and advice and free SSM being provided (or suggested) for use at the participant's discretion. Studies that used SSM as a mandatory part of the intervention protocol (including prescribed doses) were additionally coded as ‘SSM’ studies. A further three COPD-specific BCTs were identified, namely *COPD medication advice*, where advice was given regarding non-study medication that is not SSM (e.g., advice on, or optimization of COPD-related medication), *COPD-specific information,* where advice about areas of COPD management in addition to smoking cessation is given (e.g., breathing training), and *Linking COPD and smoking,* where an explicit link is drawn between the participant's smoking and their COPD (e.g., referring to the participant as having ‘smoker's lung’).

### Assessment of effectiveness and meta-analytic strategy

The effectiveness of smoking cessation interventions was assessed by two indices, the sample-weighted quit rates and *d*_*+*_. Both indices were computed using the PP and CA rates. If PP and CA rates were both reported, the outcome with the highest ranking according to Eisenberg *et al*. (2008) was used to calculate the most conservative estimate for each study. Eisenberg *et al*. ranked biochemically validated CA at 12 months most highly, followed by CA at 6 months, PP at 12 months, and finally PP at 6 months. Effect sizes were calculated using META 5.3 (Schwarzer, 1987). Random effects models were used as effect sizes from the individual studies were expected to be heterogeneous. STATA version 11 (Statacorp., 2009) was used to generate the funnel plot and to estimate small-sample bias via Egger's regression.

Potential moderators of effectiveness considered were BCTs used, study quality, study design, intervention features, type of outcome measure, and the use of SSM. For dichotomous moderators (e.g., presence vs. absence of a specific BCT), the average effect size was computed when there were at least three independent tests for both levels of the moderator, and the between-groups heterogeneity statistic (*Q*_*b*_) was used to compare the effect sizes (Webb & Sheeran, 2006). SPSS 18 (SPSS Inc., 2009) was used to compute associations between continuous moderator variables and effect sizes using weighted least squares (WLS) regression (i.e., effect sizes were weighted by the respective sample *n*).

## Results

### Studies included in the review

**Figure 1:**
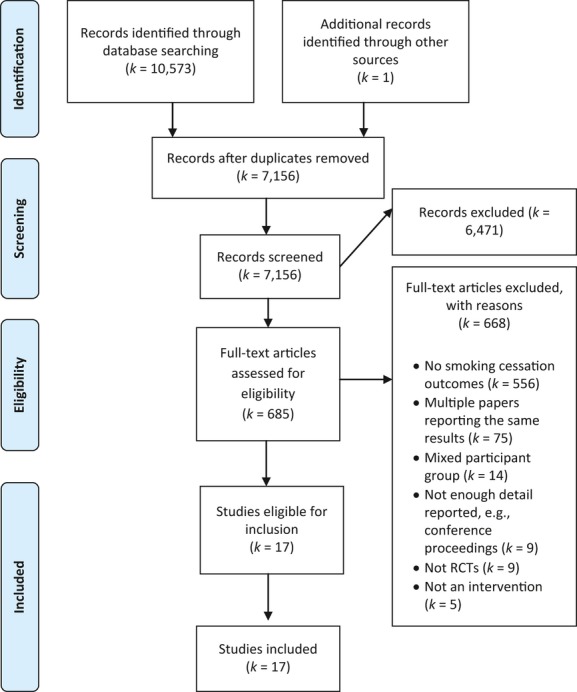
Flow of articles through the phases of the present review.

The flow of articles through the review is shown in Figure1. A total of 17 eligible interventions were identified with a total sample of 7,446 people with COPD (see Table S2, included studies are indicated in the reference list). Mean age ranged from 48 to 67 years; 42.72% of the overall sample was female (Table S3). In studies reporting FEV_1_ %pred, 2008 values ranged from 52% to 80% (*k *=* *8), which is considered moderate severity according to the GOLD (2011) standards.

The interventions mainly compared intervention conditions with usual care (*k *=* *12). Four studies were placebo-controlled drug trials, and one study compared two active conditions (Table S2). Intervention duration ranged from 22 days to 5 years (*Mdn* = 85 days; *k *=* *12). The longest follow-up (after all active components of the intervention had stopped) ranged from immediately to 2 years (*k *=* *13). The main delivery modes (*k *=* *17) were one-to-one (70.59%) or a mixture of both one-to-one and group delivery (29.41%). Intervention setting (*k *=* *17) varied between studies; 64.71% had at least some of the components delivered in the participant's home, and 35.29% were delivered exclusively in a clinical setting (Table S2).

### Quality assessment

Overall, 58.82% of studies (*k *=* *10) reached the ≥5 threshold for ‘high quality’ (van der Meer *et al*., 2003). The average quality rating overall was 5.47 (*SD *= 2.29; Table 1). Ten studies reported an *a priori* power calculation to identify a desired sample size, although only five of these studies reached their target sample size. *Post-hoc* power could be calculated for 15 studies; power ranged from 8% to 100% with an average of 63.47% (*SD *= 0.30). Ten studies reached the threshold for adequate power (55%) suggested by Coyne, Thombs, and Hagedoorn (2010). Attrition rate was the percentage of randomized participants who began the intervention, but did not complete the longest follow-up (*M = *17.46%, *SD = *10.53), where reported mean dropout during the intervention period was 16.69% (*SD *= 15.69). Overall, the quality of studies included in the review could be considered satisfactory (Table 1).

**Table 1:** Intervention outcomes

Authors	Quality Score^a^	Cessation measure^b^	Cessation criteria	A priori sample size required	Attrition rate%	*N* experimental	*N* control	Quit% experimental	Quit% control	*d*	*Post-hoc* power c
Anthonisen *et al*. (1994)	5	CA	Cotinine levels of <20 ng/mL or, if using NRT exhaled CO <10 ppm. Stopped smoking in the initial intervention and maintained this status	4000	3.5	1,961	1,964	20.80	5.20	0.48^d^	1
Borglykke, Pisinger, Jorgensen, and Ibsen (2008)	6	PP	Self-reported, validated by carbohaemoglobin <2%	NR	0	121	102	29.75	12.75	0.42	0.75
Brandt *et al*. (1997)	2	PP	Self-reported validated by CO at the final follow-up	NR	6.25	20	25	40	20	0.45	0.43
Christenhusz (2006)	4	CA	Self-reported continuous for 12 months, validated by cotinine <20 ng/mL at 6 and 12 months (must have at least 4 days abstinence for this to occur)	162	6.67	105	105	19.05	8.57	0.31	0.72
Crowley, Macdonald, and Walter (1995)	4	PP	Self-reported abstinence for 24 hr, CO <10 ppm	NR	26.53	18	15^e^	NR 15.15 total	NR 15.15 total	0^e^	NC
Efraimsson, Hillervik, and Ehrenberg (2008)	4	PP	‘Do you smoke?’ yes/no	NR	19.23^g^	16	14	37.5	0	1.06	0.88
Hilberink, Jacobs, Breteler, de Vries, and Grol (2011)	5	PP	Self-reported, did not smoke in the last 7 days, verified by urinary cotinine <50 ng/mL	300	4.3	519	148	7.51	3.38	0.14	0.56
Khdour, Kidney, Smyth, and McElnay (2009)	4^h^	CA	Self-reported as in the maintenance stage from a stage of change questionnaire at 12 months	160	17.34^g^	18	19	22.22	10.53	0.32	0.24
Kotz, Wesseling, Huibers, and van Schayck (2009)	5	CA	Abstinence at weeks 5, 26, and 52 validated by urinary cotinine <50 ng/mL	168	15.76	116	68	11.21	5.88	0.19	0.36
Pederson, Wanklin, and Lefcoe (1991)	3	CA	Self-reported quit smoking for 6 months, random sample verified by carboxyhaemoglobin levels in a blood sample	74	21.62	30	28	33.33	21.43	0.27	0.26
Sundblad, Larsson, and Nathell (2008)	3	CA	Self-reported abstinence for the last 6 months. *N* = 35 CO tested for <8 ppm	NR	18.2	192	199	38.02	10.05	0.7	1
Tashkin *et al*. (2001)	9	CA	0 cigarettes from weeks 4–26 verified at each clinic visit by exhaled CO ≤10 ppm	400	31.19	204	200	15.69	9.0	0.20	0.64
Tashkin *et al*. (2011)	9	CA	Self-reported abstinence from weeks 9 to 52, validated at each clinic visit, CO ≤10 ppm	500	33.93	248	251	18.6	5.6	0.41	1
Tønnesen, Mikkelsen, and Bremann (2006)	9	CA	Self-reported abstinence from week 2 to month 12. Verified at each clinic visit by carbon monoxide <10 ppm	268	22.16	185^i^	185^j^	14.05	5.41	0.29	0.87
Wagena, Knipschild, Huibers, Wouters, and van Schayck (2005)	9	CA	Self-reported complete abstinence from weeks 4 to 26 after quit date, confirmed by urinary cotinine of ≤60 ng/mL at weeks 4, 12 and 26 post-quit date	300	<5%^g^	96^k^	48	25^l^	8.33	0.38^m^	0.73
Wilson *et al*. (2008)	6	CA	Complete cessation for all visits. Verified by exhaled CO ≤10 ppm and salivary cotinine ≤10 ng/mL	303	25.27	56^n^	35	0	0	0^o^	NC
Zwar *et al*. (2012)	6	PP	Smoking status at 12 months, no further detail given		27.41	74	61	14.86	16.39	0.04	0.08

Ne = number in experimental group; Nc = number in control group; NR = not reported; PP = point prevalent; CA = continuous abstinence; CO = carbon monoxide; NC = not calculable; ppm = parts per million.

a Results from the Delphi list quality assessment (Verhagen *et al*., 1998)

b In studies where both CA and PP were reported, CA was used

c Calculated *post hoc* from http://www.danielsoper.com/statcalc/calc49.aspx one-tailed, using total sample size, *d,* and an alpha level of .05

d Bronchodilator versus usual care

e Control group only, excluding self-report

f Estimated, no significant difference between groups

g For whole sample

h Minimization counted as equivalent to randomization

i Sum of high and low support with NRT as there were no significant differences between groups

j Sum of high and low support with placebo as there were no significant differences between groups

k Bupropion and nortriptyline

l Total quit rate for bupropion and nortriptyline

m Combined bupropion and nortriptyline versus placebo

n Combined individual and group support groups

o Estimated 0 quit smoking in either group not reported for smokers only.

### Effectiveness of smoking cessation interventions

Individual study quit rates ranged from 0% (Wilson, Fitzsimons, Bradbury, & Elborn, 2008) to 28.9% (Brandt *et al*., 1997). The overall sample-weighted average quit rate was 13.19%. The *d*_*+*_ was 0.33 (Table 1). One study had a very large sample size and longer follow-up period compared with the other studies (Anthonisen *et al*., 1994). However, deleting this study did not significantly change the average effect size (*d*_*+*_ = 0.31) or improve homogeneity. The effect size for the nine studies with adequate power was 0.37; this value did not differ significantly from studies with inadequate power (*d*_*+*_ = 0.22). The funnel plot appeared symmetrical (Figure S1), and Egger's regression revealed no significant bias in the observed effect sizes (*B* = −1.06, *SE* = 0.58, *ns*). These findings suggest that publication bias does not present a problem for the present meta-analysis.

**Table 2:** Overall effect sizes, homogeneity, and moderator analyses

Measure	*k*	*n*	*d*_+_	95% Confidence intervals		Homogeneity analysis	
				Lower	Upper	*Q*_*w*_	*Q*_*b*_
Overall	17	7,446	0.33	0.23	0.43	41.55***	
Outliers							0.23
Excluding Anthonisen *et al*. (1994)	16	3,521	0.31	0.20	0.42	29.92*	
Quality							3.10
Adequate power and sample size^a^	9	6,833	0.37	0.27	0.48	26.7***	
Inadequate power and sample size^a^	8	613	0.22	0.00	0.43	7.28
Intervention design^b^							0.26
Drug versus placebo	4	1,417	0.31	0.21	0.42	2.61	
Intervention versus usual care	12	5,996	0.34	0.20	0.48	33.84***
Setting							13.34***
Home component	11	2,666	0.28	0.19	0.37	24.01**	
Exclusively medical setting	6	4,780	0.46	0.37	0.55	2.94
Delivery^c^							49.77***
Group components	4	4,749	0.49	0.34	0.64	6.07	
One-to-one only	12	2,606	0.26	0.14	0.38	11.86
Medication							26.23***
SSM	7	5,736	0.42	0.37	0.48	12.83*	
No SSM	10	1,710	0.32	0.13	0.50	26.48**
Outcome							3.58
PP	6	1,133	0.29	0.00	0.57	9.11	
CA	11	6,313	0.42	0.36	0.48	24.02**
BM1 ‘Provide information on the health consequences of smoking and smoking cessation’							1.87
Present	8	6,350	0.36	0.21	0.50	27.01***	
Absent	9	1,096	0.27	0.11	0.44	7.29
BM2 ‘Boost motivation and self-efficacy’							7.29**
Present	12	2,940	0.30	0.21	0.39	26.43**	
Absent	5	4,506	0.43	0.21	0.65	5.54
BM3 ‘Provide feedback on current behaviour and progress’							1.06
Present	5	4,668	0.35	0.11	0.59	16.26**	
Absent	12	2,778	0.30	0.19	0.41	11.91
BM4 ‘Provide rewards contingent on successfully stopping smoking’							0.66
Present	3	853	0.38	0.24	0.52	0.37	
Absent	14	6,593	0.32	0.19	0.44	41.03***
BM6 ‘Prompt commitment from the client there and then’							0.14
Present	3	742	0.36	0.22	0.51	1.49	
Absent	14	6,704	0.33	0.21	0.45	39.69***
BM7 ‘Provide rewards contingent on effort or progress’							2.03
Present	3	634	0.42	0.05	0.79	7.45*	
Absent	14	6,812	0.30	0.20	0.41	31.59**
BM9 ‘Conduct motivational interviewing’							0.04
Present	4	412	0.34	−0.06	0.74	5.59	
Absent	13	7,034	0.36	0.29	0.44	33.89***
BM10 ‘Identify reasons for wanting and not wanting to stop smoking’							2.78
Present	4	543	0.21	0.04	0.39	1.52	
Absent	13	6,903	0.36	0.23	0.48	34.89***
BS1 ‘Facilitate barrier identification and problem-solving’							3.00
Present	7	2,177	0.28	0.11	0.46	23.45***	
Absent	10	5,269	0.37	0.26	0.48	13.92
BS2 ‘Facilitate relapse prevention and coping’							0.17
Present	11	6,556	0.33	0.23	0.43	34.4***	
Absent	6	890	0.36	0.14	0.59	6.72
BS3 ‘Facilitate action planning/develop treatment plan’							4.72*
Present	7	5,057	0.44	0.39	0.50	11.71	
Absent	10	2,389	0.33	0.17	0.50	24.19**
BS4 ‘Facilitate goal setting’							0.17
Present	10	6,552	0.31	0.18	0.44	36.79***	
Absent	7	894	0.34	0.20	0.49	3.59
BS5 ‘Prompt review of goals’							1.08
Present	4	1,028	0.28	0.14	0.43	4.25	
Absent	13	6,418	0.35	0.23	0.48	34.17***
BS6 ‘Prompt self-recording’							4.83*
Present	5	4,962	0.40	0.22	0.57	16.10**	
Absent	12	2,484	0.29	0.15	0.42	13.86
BS8 ‘Advise on environmental restructuring’							0.14
Present	3	742	0.36	0.22	0.51	1.49	
Absent	14	6,704	0.33	0.21	0.45	39.69***
BS13 ‘Advise on methods of weight control’							33.48***
Present	3	4,539	0.53	0.37	0.69	4.35	
Absent	14	2,907	0.25	0.15	0.36	13.42
A1 ‘Advise on stop smoking medication’							0.67
Present	10	6,593	0.35	0.23	0.47	29.27***	
Absent	7	853	0.29	0.09	0.49	6.61
A2 ‘Advise on/facilitate use of social support’							8.00**
Present	7	5,372	0.40	0.23	0.57	13.40*	
Absent	10	2,074	0.25	0.11	0.39	7.99
A3 ‘Adopt appropriate local procedures to enable clients to obtain free medication’							0.15
Present	7	5,256	0.37	0.28	0.45	15.73*	
Absent	10	2,190	0.35	0.20	0.51	24.21**
A4 ‘Ask about experiences of stop smoking medication that the smoker is using’							3.45
Present	3	4,608	0.40	0.25	0.56	3.82	
Absent	14	2,838	0.31	0.18	0.43	28.12**
A5 ‘Give options for additional and later support’							0
Present	8	5,787	0.36	0.28	0.44	21.82**	
Absent	9	1,659	0.36	0.19	0.54	18.98*
RD1 ‘Tailor interactions appropriately’							3.43
Present	10	6,498	0.37	0.20	0.54	31.53***	
Absent	7	948	0.24	0.11	0.37	3.23
RD2 ‘Emphasize choice’							3.48
Present	6	5,445	0.41	0.33	0.48	10.99	
Absent	11	2,001	0.31	0.14	0.47	26.23**
RI1 ‘Assess current and past smoking behaviour’							0.03
Present	11	6,114	0.36	0.27	0.45	29.39**	
Absent	6	1,332	0.35	0.12	0.57	7.27
RI2 ‘Assess current readiness and ability to quit’							0.04
Present	8	5,300	0.35	0.25	0.45	16.25*	
Absent	9	2,146	0.36	0.21	0.51	21.94**
RI3 ‘Assess past history of quit attempts’							1.39
Present	3	774	0.26	0.05	0.48	3.90	
Absent	14	6,672	0.35	0.23	0.46	35.83***
RI5 ‘Assess nicotine dependence’							9.12**
Present	5	1,242	0.19	0.07	0.31	4.17	
Absent	12	6,204	0.39	0.27	0.51	23.67*
RI7 ‘Assess attitudes to smoking’							2.50
Present	3	485	0.21	0.03	0.39	1.47	
Absent	14	6,961	0.36	0.24	0.47	35.22***
RI10 ‘Assess physiological and mental functioning’							0.71
Present	4	4,274	0.37	−0.04	0.78	11.50**	
Absent	13	3,172	0.33	0.26	0.40	23.24*
RC1 ‘Build general rapport’							0.17
Present	4	4,410	0.32	0.14	0.50	9.15*	
Absent	13	3,036	0.34	0.21	0.46	26.83**
RC4 ‘Explain expectations regarding treatment programme’							2.07
Present	4	4,641	0.39	0.26	0.53	5.55	
Absent	13	2,805	0.32	0.19	0.44	27.34**
RC5 ‘Offer/direct towards appropriate written materials’							0.28
Present	9	5,872	0.41	0.36	0.46	21.77***	
Absent	8	1,574	0.38	0.19	0.58	19.58**
RC6 ‘Provide information on withdrawal symptoms’							2.41
Present	6	1,535	0.39	0.24	0.54	12.75*	
Absent	11	5,911	0.30	0.16	0.44	28.78**
RC8 ‘Elicit client views’							0.36
Present	5	1,042	0.31	0.19	0.43	3.93	
Absent	12	6,404	0.35	0.22	0.49	34.87***
COPD medication advice							0.02
Present	4	247	0.37	−0.03	0.76	5.82	
Absent	13	7,199	0.35	0.26	0.43	34.44***
COPD-specific information							1.35
Present	7	1,489	0.35	0.11	0.59	23.61***	
Absent	10	5,957	0.42	0.37	0.47	16.15
Link between COPD and smoking							8.42**
Present	4	4,524	0.45	0.39	0.51	6.11	
Absent	13	2,922	0.31	0.18	0.45	28.92**
BS ‘Specific focus on behaviour, maximizing self-regulatory capacity/skills’							1.13
Present	13	7,190	0.32	0.23	0.42	38.92***	
Absent	4	256	0.46	0.18	0.75	2.50
A ‘Promote adjuvant activities’							0.89
Present	13	7,169	0.33	0.24	0.42	38.72***	
Absent	4	277	0.45	0.14	0.76	2.79

Note. SSM = stop smoking medication.

a Defined as power >0.5 and sample size ≥35 in each cell (Coyne *et al*., 2010)

b One study compared two active conditions

c *k *= 15 Wilson *et al*., excluded as their experimental groups compared individual and group support.

* Significant at *p *<* *.05

** significant at *p *<* *.01

*** significant at *p *<* *.001.

Potential moderators of the observed effect sizes were tested using the *Q*_*b*_ statistic (Table  2008). The effect size for measures of CA (*k *=* *11, *d*_*+*_ = 0.42) was higher than those studies reporting only PP (*k *=* *6, *d*_*+*_ = 0.29), but the difference did not reach significance (*Q*_*b*_ = 3.58, *p = *.06). The nature of the comparison group (usual care vs. placebo) did not influence effect sizes (Table 1). However, the provision of SSMs and both delivery and setting of the interventions were significant moderators. Interventions that provided SSM as a mandatory part of their protocol (*k *=* *7, *d*_*+*_ = 0.42) were more effective than interventions that did not (*k *=* *10, *d*_*+*_ = 0.32), *Q*_*b*_ = 26.24, *p* < .001. Interventions delivered exclusively in a clinical setting (*k *=* *6, *d*_*+*_ = 0.37) had a significantly higher *d*_*+*_ than those that contained either home components, or were delivered exclusively at home (*k = *11, *d*_*+*_ = 0.28), *Q*_*b*_* *= 13.34, *p* < .001. Interventions containing group components (*k *=* *4, *d*_*+*_ = 0.49) had a significantly higher effect size than one-to-one-only interventions (*k *=* *12, *d*_*+*_ = 0.26), *Q*_*b*_ = 49.77, *p* < .001. Potential continuous moderators were entered into WLS regressions. Study quality (*k = *17, *β *= .27, *p *=* *.30), duration of the intervention (*k *=* *13, *β *= .48, *p *=* *.10), the time between the end of the intervention to the longest follow-up (*k *=* *13, *β *= .07, *p *=* *.82), and attrition rate (*k *=* *17, *β *= −.245, *p *=* *.34) did not significantly predict effect sizes.

### BCTs and intervention effectiveness

Of the 53 smoking cessation BCTs identified by Michie, Churchill, *et al*. (2011), 47 were used in one or more of the interventions. The number of techniques used in each intervention ranged from 1 to 28, with an average of 13.11 (*SD *= 8.63; Table S2). The most frequently used individual technique was *Boost motivation and self-efficacy*, which was used in 70.59% of the interventions.

The impact of presence versus absence of particular BCTs on effectiveness was tested using the *Q*_*b*_ statistic (Table 1). Two techniques were associated with reduced effectiveness (*Boost motivation and self-efficacy* and *Assess nicotine dependence*). However, there were positive effects for four techniques: Interventions that deployed *Facilitate action planning/develop treatment plan*, *Prompt self-recording*, *Advise on methods of weight control*, and *Advise on/facilitate use of social support* each engendered significantly larger effect sizes compared with studies that did not use these techniques.

Two groups of BCTs had ≥3 studies in both presence and absence levels to be analysed. Interventions that used BCTs focussing on self-regulatory capacity/skills (BS codes) and interventions that promoted adjuvant activities (A codes) were compared with interventions that did not; neither comparison was significant (*Q*_*b*_* *= 1.13, *p *=* *.29 and *Q*_*b*_* *= 0.89, *p *=* *.34 respectively). The provision of *COPD-specific information* or *COPD medication advice* was not associated with effect sizes (*Q*_*b*_* *= 1.35, *p *=* *.25 and *Q*_*b*_* *= 0.02, *p *=* *.88, respectively). However, interventions that involved *Linking COPD and smoking* generated larger effect sizes (*Q*_*b*_* *= 8.42, *p *<* *.01).

## Discussion

Seventeen RCTs of smoking cessation interventions for people with COPD were identified. The sample-weighted average quit rate across these trials was 13.19%. This rate is higher than the 5% expected quit rate for general population smokers with no help (Hughes, Keely, & Naud, 2004), marginally higher than the 12.3% quit rate reported in a previous review of people with COPD (Hoogendoorn *et al*., 2010)**,** and lies within the range of general population quit rates in response to behavioural interventions for smoking (Poulsen, Dollerup, & Moller, 2010). It has been reported that people with COPD find it harder to quit than the general population of smokers (Tashkin & Murray, 2009), so it was expected that the quit rate observed here (13%) falls below the minimum expected quit rate of 35% in the SSS (Willis, 2008) and below the actual quit rate of 49% achieved by NHS SSS across England in 2011/2012 (The Health and Social Care Information Centre, 2012). It is notable that no statistics are available for quit rates for people with COPD through the SSS. However, the relatively high prevalence of COPD suggests that even a quit rate of 13% would be important for health care services (Tashkin & Murray, 2009). The magnitude of the sample-weighted average effect size is also consistent with the idea that smoking cessation interventions for people with COPD were generally effective. The effect size observed here (*d*_*+*_ = 0.33) is in the modal range obtained in a review of 302 meta-analyses of psychological and behavioural treatments (Lipsey & Wilson, 1993).

Interventions containing group elements and those delivered within a clinical setting were found to be effective in this population. The increased benefit of including group elements, over and above individual counselling, in smoking cessation interventions for the general population is currently unclear (Stead & Lancaster, 2009). Further research is needed to ascertain whether this approach is more effective for people with COPD than the general population of smokers. Smoking cessation interventions delivered while patients are hospitalized, with a range of conditions, and containing follow-up extending beyond the period of hospitalization have been found to be more effective than usual care in a meta-analysis (Munafò, Rigotti, Lancaster, Stead, & Murphy, 2001). Clinical settings are smoke-free environments, and all other cues to smoking associated with being in the home would be removed in these interventions; these additional factors may have contributed to interventions delivered in a clinical setting being more effective for people with COPD.

A novel feature of the present meta-analysis was that the BCTs used in smoking cessation interventions for people with COPD were coded, and their impact on effectiveness was tested. Across all 17 interventions, four established BCTs were associated with significantly larger effect sizes: *Facilitate action planning/develop treatment plan*, *Prompt self-recording*, *Advise on methods of weight control*, and *Advise on/facilitate use of social support*. In addition, one new COPD-specific BCT *Linking COPD and smoking* was also found to be associated with larger effect sizes. Forming detailed plans of what, when, and how to achieve a behaviour change have been found to be effective in achieving a wide range of behaviour change targets (Gollwitzer & Sheeran, 2006). Implementation intentions take the format of if–then plans and have been found to be effective not only in promoting initial changes in behaviour (Sheeran & Orbell, 1999), but also in protecting ongoing behavioural performance from antagonistic feelings and cognitions (Achtziger, Gollwitzer, & Sheeran, 2008; Martin, Slade, Sheeran, Wright, & Dibble 2011). The current findings suggest that: prompting the formation of if–then plans, providing information about how to handle weight gain as a possible side effect of cessation, and facilitating self-monitoring of current behaviour and progress towards the goal could each constitute useful components of smoking cessation interventions for this population.

The finding that *Advise on/facilitate use of social support* was associated with more effective smoking cessation interventions for people with COPD echoes the results of a previous review concerning smoking cessation in the general population (West *et al*., 2010). However, eight techniques that West *et al*. found were effective in the general population (and were used in ≥3 tests in the present review) were not associated with effect sizes here. These findings suggest that although fewer techniques are effective for people with COPD than for members of the general public, social support is an important aid to quitting for all smokers. Such findings also imply that it may be advantageous to tailor smoking cessation efforts to the target sample as it cannot be assumed that BCTs that are effective for members of the general public are similarly effective for specific groups.

Two techniques, *Assess nicotine dependence* and *Boost motivation and self-efficacy*, were contraindicated among smokers with COPD. One possible explanation for the negative effect of assessing nicotine dependence is that such assessment could reinforce the idea that the person is ‘addicted’ to smoking and thus reduce self-efficacy in relation to quitting. Further primary research on how best to feed back nicotine dependence assessments is needed to test this hypothesis. A possible explanation for the second contraindicated BCT is that smokers with COPD who take part in smoking cessation interventions may already be highly motivated to quit. Additional attempts to boost motivation and self-efficacy could therefore lead to *overmotivation*, which is known to hamper effective goal striving and undermine rates of goal attainment (Baumeister, 1984; Heckhausen & Strang, 1988). Consistent with this idea, none of the BCTs that concerned improving motivation (i.e., BM codes in Michie, Hyder, *et al*.'s 2011 taxonomy) proved effective. It has previously been reported that smokers with COPD may fall into two motivational categories, namely those who are unmotivated to quit and would benefit from motivational techniques, and participants who are motivated to quit and would benefit from volitional interventions such as implementation intentions (Hilberink, Jacobs, Schlosser, Grol, & de Vries, 2006). It may be important, therefore, to tailor interventions appropriately. This review suggests that, for smokers with COPD who participated in these interventions, building self-regulation capacity and skills that facilitate the translation of motivation into action may be more important than techniques aimed at merely increasing motivation to quit smoking. The implication is that future studies would do well to measure motivation and self-regulation capacity prior to conducting the intervention, so that time and resources can be devoted to the particular issues faced by participants (forming strong intentions to quit and/or the effective implementation of quit intentions).

### Limitations and directions for future research

The main limitation of the present review is the paucity of RCTs that were available for analysis (*k *=* *17). The quality of the included studies was variable, with 7 of 17 falling below the threshold for ‘high quality’ (van der Meer *et al*., 2003). Furthermore, only 10 studies reported an *a priori* power calculation, and only 9 studies were adequately powered according to Coyne *et al*.'s (2010) criteria. Although both the funnel plot and Egger's regression suggest that publication bias does not present a problem for this review, including unpublished or grey literature may have allowed a larger sample of RCTs to be considered. In future, the inclusion of high-quality grey literature should be considered.

Descriptions of the BCTs used in interventions in the original articles were often brief, and while efforts were made to retrieve further information, the full range of BCTs deployed may not have been captured in all studies. It has been reported recently that fewer than one-half of the BCTs listed in intervention manuals and protocols are reported in the final publications (Lorencatto, West, Stavri, & Michie, 2012). The introduction of online supplements and requiring submission of the full intervention protocol before publication of RCTs should mean that reports of protocols will improve in future, although Lorencatto, West, Stavri, *et al*. (2012) have not found evidence of this improvement thus far. A related difficulty is that there is no way of knowing whether all of the reported BCTs were actually delivered during the intervention. Finally, the large number of moderators considered introduces the potential of some being significant by chance. To address this issue, the higher-level categories within the Michie, Hyder, *et al*.'s (2011) taxonomy (motivation, self-regulatory capacity/skills, adjuvant activities, and general aspects of the interaction) were also considered. This approach was taken in a previous review identifying effective approaches to increase exercise-related self-efficacy (Ashford, Edmunds, & French, 2010). However, although the majority of interventions in the present review included techniques from all four categories, only two categories reached the necessary *k *≥* *3 tests in both the absence and presence categories. These limitations are inherent to coding BCTs from a small number of published reports. Analysis with a greater number of primary studies, specifically investigating the roles of motivating and self-regulating BCTs for people with COPD, and how these techniques are being delivered would consolidate these initial results and allow for more confidence in designing smoking cessation interventions for this population. Additional RCTs of smoking cessation interventions for people with COPD should be a priority in future research (Parker & Eaton, 2012).

Additional studies are needed to permit more powerful tests of the effectiveness of BCTs and more authoritative analyses of the specific BCTs that engender the greatest cessation rates. As current UK practice is to refer people with COPD to the SSS, any new interventions should be evaluated in relation to the quit rates observed in the SSS. Future studies also need to be adequately powered and whenever possible should adopt the ‘Russell Standard’ for the measurement of smoking cessation (6- or 12-month biochemically validated abstinence; West, Hajek, Stead, & Stapleton, 2005). Finally, reports of RCTs should follow the CONSORT recommendation (Schulz, Altman, & Moher, 2010) that all intervention procedures are described ‘with sufficient details to allow replication’ (p. 699) to facilitate cumulative knowledge concerning the effectiveness of BCTs.

### Conclusions

This meta-analysis aimed to identify the most effective BCTs used in smoking cessation interventions for people with COPD. Seventeen RCTs were identified, a mean quit rate of 13.19% and a sample-weighted average effect size of 0.33 were observed. Two BCTs were contraindicated, and five BCTs were associated with improved effectiveness. The present findings suggest that boosting motivation and assessing nicotine dependence may be counterproductive, whereas facilitating action planning, prompting self-recording, offering advice on weight control and the use of support, and linking COPD and smoking should each prove helpful in future smoking cessation interventions for people with COPD. Further research, including studies investigating interventions tailored according to an individual's initial motivation and self-regulatory capacity, is needed to corroborate the findings obtained here. More and better-quality studies will help to identify the most effective BCTs and so ensure that smoking cessation interventions for people with COPD are as effective as possible.

## 1. Appendix

### Full search strategy

1. Lung disease*, obstructive (mapped to subject heading where applicable).
2. Pulmonary disease, chronic obstructive (mapped to subject heading, exploded if applicable).
3. Emphysema*.
4. (chronic adj3/N3 bronchitis*).
5. (obstruct* adj3/N3 (lung* or airway* or airflow* or bronch* or respirat*)).
6. COPD or COAD or COBD or AECB.
7. 1 or 2 or 3 or 4 or 5 or 6.
8. Exercise or ‘exercise movement therapy’ or ‘exercise therapy’ or kinisio*therapy.
9. (physical or exercise)adj/N1 (train* or fitness or activit* or therap*).
10. 8 or 9.
11. Abstain* or smok* or giv* or tobacco* or nicotine* or anti*smoking or quit* or stop* or cessat* or ceas* or abstin*.
12. Pursed lip breath* or diaphragm* breath or breath* or inspiri* or ‘ventilation feedback training’ or yoga or ‘chest physiotherapy’ or ‘chest physical therapy’.
13. Behav* or intervention*.
14. 10 or 11 or 12.
15. 7 and 14 and 13.

## Supporting Information

The following supporting information may be found in the online edition of the article

Table S1. Functional classification of the smoking cessation taxonomy, as described in the supplementary material for Michie, Churchill *et al*. (2011). **Table S2**. Intervention characteristics. **Table S3**. Sample characteristics. **Figure S1.** Funnel plot.
