# Supplementary material for: Effective behaviour change techniques in smoking cessation interventions for people with chronic obstructive pulmonary disease: A meta-analysis
Source: Br J Health Psychol. 2013 Oct 30;19(1):181–203. doi: 10.1111/bjhp.12071 (PMC4253323; doi:10.1111/bjhp.12071)
Supplement: Table S1. — Functional classification of the smoking cessation taxonomy, as described in the supplementary material for Michie, Churchill et al. (2011). Table S2. Intervention characteristics. Table S3. Sample characteristics. Figure S1. Funnel plot. [file bjhp0019-0181-SD1.docx]

Supplementary Material for:

**Effective behaviour change techniques in smoking cessation interventions for people with chronic obstructive pulmonary disease (COPD): A meta-analysis**

Yvonne Kiera Bartlett^1^, Paschal Sheeran^2^ and Mark S. Hawley^1^

^1^School of Health and Related Research, University of Sheffield

^2^Department of Psychology, University of Sheffield

**Table S1** *Functional classification of the* *smoking cessation taxonomy, as described in the supplementary material for Michie, Churchill & West (2011)*

| *Functional Classification* | | *Example BCT* | *Example Description* |
| --- | --- | --- | --- |
| (B) Specific focus on behaviour | (M) Addressing Motivation | BM1: Provide information on the consequences of smoking and smoking cessation | Give, or make more salient, information about the harm caused by smoking and the benefits of stopping; distinguish between the harms from smoking and nicotine; debunk myths about low tar and own-roll cigarettes and cutting down |
|  |  | BM4: Provide rewards contingent on succesfully stopping smoking | Give praise or other rewards if the person has not smoked |
|  | (S) Maximising self-regualtory capacity/ skills | BS1: Facilitate barrier identification and problem solving | Help the smoker to identify general barriers (e.g. susceptibility to stress) that might make it harder to stay off cigarettes and develop general ways of addressing these |
|  |  | BS4: Facilitate goal setting | Help the smoker to set a quit date and goals that support the aim of remaining abstinent |
| 1. Promote adjuvant activities |  | A1: Advise on stop-smoking medication | Explain how the benefits of medication, safety, potential side effects, contra-indications, how to use them most effectively, ad how to get them; advise on the most apprpriate medication for the smoker and promote effective use |
|  |  | A4: Ask about the experiences of stop smoking medication that the smoker is using | Assess usage, side effects and benefits experienced of medication(s) that the smokeris currently using |
| (R) General aspects of the interaction | (D) Focusing on the delivery of the intervention | RD1:Tailor interactions appropriately | Use relevant information from the client to tailor the behavioural support provided |
|  |  | RD2: Emphasise choice | Emphasise client choice within the bounds of evidence based practice |
|  | (I) Focusing on information gathering | RI1: Assess current and past smoking behaviour | Assess amount smoked, age when started, pattern of smoking behaviour |
|  |  | RI2: Assess current readiness to quit | Assess amount smoked, age when started, pattern of smoking behaviour |
|  | (C) Focusing on general communication | RC1: Build general rapport | Establish a postive, friendly and professional relationship with the smoker and foster a sense that the smoker’s experiences are understood |
|  |  | RC4: Explain expectations regarding the treatment programme | Explain to the smoker the treatment programme, what it involves, the active ingredients and what it requires of the smoker |

**Table S2** *Intervention Characteristics*

| *Reference* | *Country* | *BCTs used in intervention* | *BCT category (n)* | *SSM* | *Comparison* | *Duration* | *Longest follow-up* | *Session description* | *Delivery* | *Setting* |
| --- | --- | --- | --- | --- | --- | --- | --- | --- | --- | --- |
| Anthonisen et al.,1994^1^ | USA | BM1, BM3, BM12 BS2, BS3, BS4, BS6, BS10, BS13, A1, A2, A3, A4, A5, RD1, RD2, RI1, RI2, RI10, RC1, RC4, RC5, COPD Med, COPD Spec | 4 | Yes | Usual Care | 5 years | 5 years | 1 meeting with physician,1 orientation meeting, 12 group meetings over 10 weeks, 4 per week, then declining in frequency. Minimum monthly maintenance visits until participant remained abstinent for 8 months (through 2 follow-up visits). 15 follow-up visits, every 4 months for 5 years. Further opportunities to attend sessions as needed. | One to one and group | Clinic |
| Borglykke et al.,2008 ^2^ | Denmark | BM1, BM2, BM11, BS2, BS3, BS4, BS13, A1, A2, A3, A5, RD1, RD2, RI2, RI5 | 4 | No | Usual Care | 5 weeks | 1 year | 5 weekly group sessions of 2 hours | Group | Hospital |
| Brandt et al.,  1997 | Denmark | BM2, RI1, COPD med, COPD spec, COPD/smoke link | 2 | No | Usual Care | NR | 1 year | Every time the medical staff spoke to the participant about their illness in the time they were at hospital, and all the materials they received | One to one | Hospital |
| Christenhusz et al.,2006 | Netherlands | BM2, BM4, BM6, BM7, BM9, BM10, BS1, BS2, BS5, BS8, BS11, BS14, A1, A2, A3, A5, RD1, RD2, RI2, RI7, RC1, RC8, COPD spec | 4 | Yes | Usual Care | NR | 1 year | Four small-group meetings (total 6 hours), four individual sessions (total 195 minutes), four telephone contacts (total 40 minutes) | One to one and group | Home. Unclear for group sessions |
| Crowley et al.,1995 | USA | BM2, BM3, BM6, BM7, BM12, BS2, BS3, BS4, BS8, A1, A2, A3, RI1, RI2, RI5, RC4,RC3, RC5,RC6 | 4 | No^7^ | Rewarded but dependant on a yoked control’s smoking behaviour, not their own. | 86 days | 6 months | On each of 86 days, a researcher guided by manual visited the participants’ home. | One to one | Home |
| Efraimsson et al., 2008 | Sweden | BM9, RD1, RI10, COPD med, COPD spec | 2 | No | Usual Care | 3-5 months | 3-5 months | 2 visits to the COPD clinic, 2 visits for self-care education with a nurse. | One to one | Clinic |
| Hilberink et al.,2011 | Netherlands | BM1, BM2, BS1, BS2, BS4, A1, A5, RD1, RI5, RC5, COPD spec. | 4 | No^8^ | Usual Care | NR | 1 year | Unclear (dependant on motivational stage, maximum of 2 follow-up visits and 3 follow-up phone calls) | One to one | GP practice and home |
| Khdour et al.,2009 | UK | BM9, A5, RI1,COPD med, COPD spec | 3 | No | Usual Care | 1 year | 1 year | 3 outpatient clinic appointments and 2 phone calls | One to one | Pharmacy and Home |
| Kotz et al. , 2009^3^ | Netherlands | BM1, BM2, BM3, BM10, BS1, BS2, BS3, BS4, BS5, BS6, A1, A4, RD2, RI1, RI2, RI3, RI5, RI7, RI10, RC1, RC2, RC4, RC5, RC6, RC7, RC8, RC9, RC10, COPD smoking link | 4 | Yes | Usual care^9^ | 22 days | 1 year | 4*40 minute sessions 3 face to face and 1 telephone | One to one | Clinic and home |
| Pederson et al.,1991 | USA | BM2, BM10, BS2, BS3, BS6, RD1, RI9, RC2, RC5, RC6, RC8 | 3 | No | Usual Care | Duration of hospital stay | 6 months | 1 initial, then 2 to 8 follow-up sessions while at hospital. | One to one | Hospital |
| Sundblad et al.,2008 | Sweden | BM1, BM2, BM3, BM7 ,BS1, BS3, BS4 BS6, BS13, A1, A2, RD1, RI1, RI9, RC6, COPD spec. | 4 | No | Usual care | 1 year | 3 years | 11 hours with a smoking cessation nurse, then education sessions during one 2-week period of admission, a further 2 - 4 days admission if agreed to. Then 24-28 follow-up phone calls lasting 5 - 30 minutes | One to one and group | Hospital and Home |
| Tashkin et al., 2001 | USA | BM2, BS2, BS4, BS6, BS8, A3, RD1, RD2, RI1 | 4 | Yes | Placebo | 12 weeks | 6 months | 9 face-to-face sessions at clinic visits, 1 telephone counselling, diaries completed everyday so a further 84 self-directed sessions | One to one | Clinic and Home |
| Tashkin et al., 2011^5^ | USA/Spain/France & Italy | BM1, BM2, BM4, BM6, BM8, BM11, BS1, BS3, BS4, BS5, BS7, BS11, A1, A2, A4, A5, RD1, RD2, RI1, RI2, RI3, RC4, RC5, RC6, RC8, RC10 | 4 | Yes | Placebo | 1 year | 1 year | In the 12-week treatment phase participants visited the clinic 12 times (weekly) and were telephoned once; in the follow-up phase (no pharmacological treatment, brief counselling at every visit and phone call) participants visited the clinic 7 times and were telephoned 5 times. | One to one | Clinic and Home |
| Tønnesen et al.,2006 | Denmark | BM1, BS2, A1, A3, RI1, RC5, RC6, COPD smoke link | 4 | Yes | Placebo | 12 weeks | 1 year | Low support condition: 4*20-30-min visits, 6*10-min phone calls.  High support condition:7*20-30-min visits, 5*10-min phone calls. | One to one | Clinic and Home |
| Wagena et al., 2005 | The Netherlands | BM4 | 1 | Yes | Placebo | 12 weeks | 26 weeks | Baseline visit, 10-20 minutes face-to-face counselling at weeks 1, 3 and 12 post quit date. Supportive telephone calls on the quit date and 2, 5, 6, 8 and 11 weeks post quit date. | One to one | Clinic and Home |
| Wilson et al., 2008 ^4^ | UK | BM1, BM2, BM10, BS1, BS2, BS4, A1, A2, A3, A5, RD1, RI1, RI2, RI3, RI7, RC1, RC5, RC8 | 4 | No | Usual Care | 5 weeks | 1 year | Up to a maximum of 5*60min sessions, depending on the experimental group | One to one or group depending on the experimental condition | Clinic |
| Zwar et al., 2012^6^ | Australia | BM2, BM3, BM9, BS1, BS3, BS4, BS5, A5, RI1, RI2, RI5, RI10 | 4 | No | Usual Care | 26 weeks | 1 year | Initial home visit with spirometry. Collaborative work with nurse, GP and patient to implement the plan, including at least 2 home visits, 5 phone call and two GP visits. Including referring to external sources for pulmonary rehab and smoking cessation. | One to One | GP Practice and Home |

*Note:* Additional BCT information coded from: ^1^ (O'Hara, Grill, Rigdon, Connett et al., 1993) ^2^ (Kjaer, Evald, Rasmussen, Juhl et al., 2007) ^3^ (Kotz, Wesseling, Huibers, & van Schayck, 2007)^4^ (Raw, McNeill, & West, 1998)^5^(Fiore, Jaen, & Baker, 2008).^6^ Additional information from online supplement, email response and (Zwar, Hermiz, Hasan, Comino et al., 2008) ^7^NRT was given to both intervention and control group, used at participants discretion, not a mandatory part of the intervention ^8^Although [45] stated provision of SSM was part of their protocol, they reported that very few participants took the medication suggested, as this was reported this study has been coded as not containing SSM ^8^ Non-confrontational counselling group not reported here BM1: Provide information on the health consequences of smoking and smoking cessation; BM2: Boost motivation and self-efficacy; BM3: Provide feedback on current behaviour and progress; BM4: Provide rewards contingent on not smoking; BM6: Prompt commitment from the client there and then; BM7: Provide rewards contingent on effort or progress; BM8: Strengthen ex-smoker identity; BM9:Conduct motivational interviewing BM10: Identify reasons for wanting and not wanting to stop smoking; BM11: Explain the importance of abrupt cessation; BM12: Measure carbon monoxide (CO); BS1: Facilitate barrier identification and problem solving; BS2: Facilitate relapse prevention and coping; BS3: Facilitate action planning/ develop a treatment plan; BS4: Facilitate goal setting; BS5: Prompt review of set goals; BS6: Prompt self-recording; BS7:Advise on changing routine; BS8: Advise on environmental restructuring; BS10: Advise on conserving mental resources; BS11:Advise on avoidance of social cues for smoking; BS13: Advise on methods of weight control; BS14: Teach relaxation techniques; A1: Advise on stop smoking medication; A2: Advise on/facilitate use of social support; A3: Adopt appropriate local procedures to enable clients to obtain free medication ; A4: Ask about experiences of stop smoking medication that the smoker is using ;A5: Give options for additional and later support; RD1: Tailor interactions appropriately; RD2: Emphasise choice; RC1: Build general rapport; RC2: Elicit and answer questions; RC3: Explain the purpose of carbon monoxide monitoring ; RC4: Explain expectations regarding treatment programme ; RC5: Offer/direct towards appropriate written materials; RC6: Provide information on withdrawal symptoms; RC7: Use reflective listening; RC8: Elicit client views ; RC9: Summarise information / confirm client decisions; RC10: Provide reassurance; RI1: Assess current and past smoking behaviour; RI2: Assess current readiness and ability to quit ;RI3: Assess past history of quit attempts; RI4: Assess withdrawal symptoms; RI5: Assess nicotine dependence; RI6: Assess number of contacts who smoke; RI7: Assess attitudes to smoking; RI9: Explain how tobacco dependence develops; RI10: Assess physiological and mental functioning.

**Table S3** *Sample Characteristics*

|  |  | *Mean (SD) Age* | | *Mean (SD)FEV_1_% predicted*^†^ *at baseline* | |
| --- | --- | --- | --- | --- | --- |
| *Authors* | *Female %* | *Experimental* | *Control* | *Experimental* | *Control* |
| Anthonisen et al.,1994 | 37.13 | 48.4 (6.8) ^a^ | 48.4 (6.9) | 75.1%^a^(8.8) | 75.1% (8.8) |
| Borglykke et al.,2008 | 64.57 | 65 (NR) | 67 (NR) | NR | NR |
| Brandt et al.,1997 | NR | 66 (range 38-88)^b^ | 66 (range 38-88)^b^ | NR | NR |
| Christenhusz et al.,2006 | 47.56 | 59.6 (8.51) | 57 (8.41) | 62.8 (25.7) | 65.6 (27.4) |
| Crowley et al.,1995 | 24.24 | 62.3 (NR) ^c^ | 63 (NR) | NR | NR |
| Efraimsson et al., 2008 | 50 | 66 (9.4) | 67 (10.4) | NR | NR |
| Hilberink et al.,2011 | 50.97 | 60.7(11.2)^d^ | 60.1 (11.5)^f^ | NR | NR |
| Khdour et al.,2009 | 56.1^e^ | 65.63(10.1)^e^ | 67.3 (9.2)^e^ | 52(15.9)^e^ | 52(17.8)^e^ |
| Kotz et al. , 2009 | 39.67 | 53.8 (7.0) | 53.0 (7.6)^f^ | 80.5(14.7) | 79.7(14.0)^f^ |
| Pederson et al.,1991 | 31.08 | 53.4 (13.7)^b^ | 53.4 (13.7)^b^ | NR | NR |
| Sundblad et al.,2008 | 50.34 | 53 (range 41-62) | 52 (range 41-61) | 74(16) | 77(14) |
| Tashkin et al., 2001 | 45.01 | 53.2 (9.0) | 54.5 (9.5) | 73.2(19.4) | 69.4(17.3) |
| Tashkin et al., 2011 | 37.68 | 57.2(9.1) | 57.1(9.0) | 70.8(17.0) | 69.1 (16.9) |
| Tønnesen et al.,2006 | 52.16 | 59.2 (10.3)^g^  61.3 (9.6)^i^ | 62.5(9.3)^h^  61.2 (9.4)^j^ | 73.4(17.5)^g^  71.3(19.2)^i^ | 73.8(20.9)^h^  73.1(16.4)^j^ |
| Wagena et al., 2005 | 51(8.5)^e^ | 51.1(8.3)^k^  51.2(9.1)^l^ | 51.3 (8.4)^k^ | NR | NR |
| Wilson et al., 2008 | 51.65 | 61(84)^b^ | 61(84)^b^ | NR | NR |
| Zwar et al., 2012 | 52.11^e^ | 65.8 (10.3)^e^ | 64.4(10.3)^e^ | NR | NR |

Note: ^†^Forced Expiratory Volume in one second, presented as a % of what would be expected for someone of the same age, gender and height (FEV_1_%pred) ^a^ Bronchodilator vs. Usual care; ^b^ Group ages not reported, average across groups; ^c^ Experimental and Control; ^d^ Counselling, Nicotine replacement and Buproprion Group; ^e^ For the whole sample, not only smoking people with COPD; ^f^ Usual care ^g^ Nicotine and low support; ^h^ Placebo and low support; ^i^ Nicotine and High support; ^j^ Placebo and high support; ^k^Bupropion ; ^l^ Nortriptyline; ^k^ Placebo; NR= Not Reported.


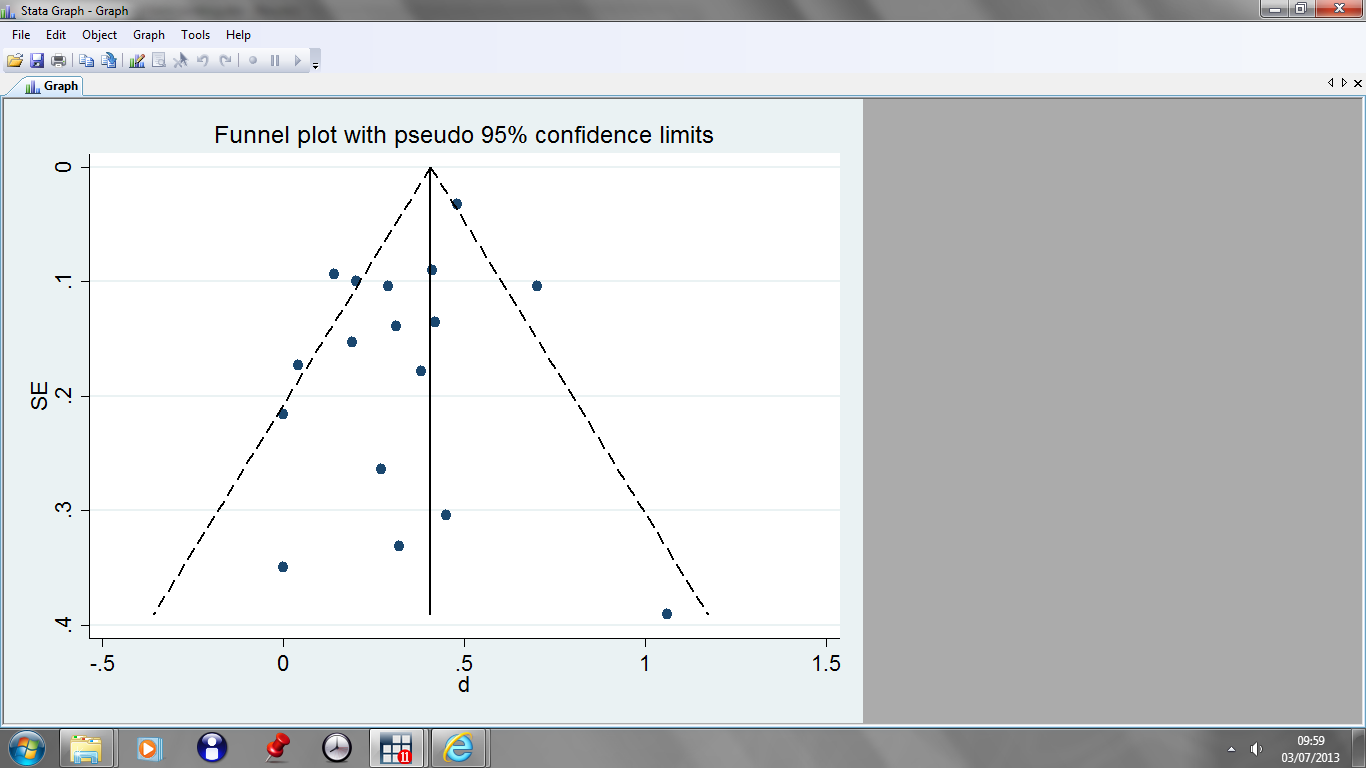


**Figure S4.** *Funnel Plot*
